# Iron-Based Adsorbents Derived from Groundwater Deferrization Sludge for Spent Oil Removal from Aqueous Media

**DOI:** 10.1021/acsenvironau.5c00217

**Published:** 2025-11-17

**Authors:** Valentin Romanovski, Alesia Harelaya, Haitao Wang, Dmitry Moskovskikh

**Affiliations:** † Department of Materials Science and Engineering, 2358 University of Virginia, Charlottesville, Virginia 22904, United States; ‡ Department of Water Supply, Chemistry and Ecology, Belarusian State University of Transport, 34 Kirova Street, Gomel 246653, Belarus; § College of Environmental Science and Engineering, 12538 Nankai University, Tianjin 300350, China; ∥ Science and Research Centre of Functional Nano-Ceramics, National University of Science and Technology “MISIS”, Moscow 119049, Russia

**Keywords:** Fe_*x*_O_*y*_, nanoparticles, sorption, oil products, waste recycling

## Abstract

The paper presents the results of the synthesis and study of iron oxide-based sorbents (Fe_*x*_O_*y*_-NPs) obtained from iron removal station sludge by exothermic combustion in solution using glycine, urea, citric acid, and urotropine as reducing agents. X-ray phase analysis revealed that the phase composition depends on the nature of the reducing agent and temperature: at 300–500 °C, the magnetite content reached 97–99% for citric acid and urea, whereas when using glycine, the Fe_3_O_4_ fraction did not exceed 30%. The point of zero charge values shifted to the alkaline region with increasing synthesis temperature, reaching 8.8 at 700 °C. The specific surface area for methylene blue was up to 186 m^2^/g, but the calculated values exceeded the BET data by 3.5–4 times due to multilayer sorption on the functionalized surface, which is consistent with the FTIR spectra. The oil sorption capacity (OSC) of the synthesized sorbents reached 6.1 g/g (glycine, 500 °C), which is comparable to or exceeds the indicators of a number of natural and commercial sorbents. After five sorption-regeneration cycles at 800 °C, the OSC decreased by only 15.7%, confirming the stability of the material. The constructed polynomial and machine learning models (CatBoost, XGBoost) provided high accuracy of OSC prediction (*R* ^2^ = 1.0), which demonstrates the promise of machine learning for optimizing synthesis conditions.

## Graphical abstract

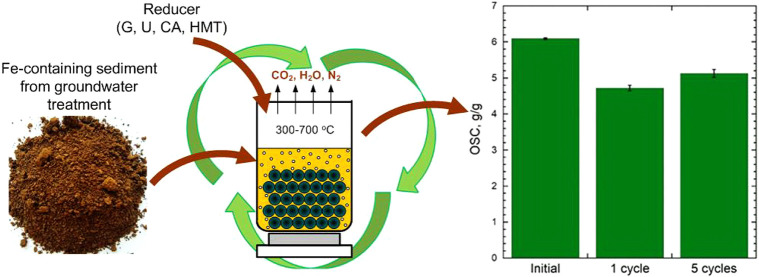

## 1 Introduction

Modern approaches to water resource protection include the development of effective methods for the removal of persistent organic pollutants, such as pharmaceutical residues,^1,2^ petroleum compounds,^3,4^ and aromatic hydrocarbons,^5,6^ as well as heavy metal ions.^7,8^ At the same time, an important area is the processing of industrial and municipal waste to obtain functional materials for water purification, in particular, adsorbents^9,10^ and photocatalysts.^11–15^ These initiatives are in line with the priorities of the Sustainable Development Goals (SDGs), especially SDG 6, aimed at ensuring access to safe water, and SDG 12, related to sustainable production cycles. Water treatment waste, including spent ion exchange materials,^16,17^ iron-containing filtration residues,^18,19^ and coagulation sludges,^20,21^ is of interest as a raw material for obtaining valuable materials. When using iron removal filters in water supply systems, sediments enriched with iron compounds are formed.^22–25^ These materials, previously considered primarily as waste, have recently been studied as a source for the synthesis of multifunctional inorganic compounds with the possibility of their use in water purification. Industrial and municipal wastewater often contains petroleum products, including waste oils, diesel fuel, lubricants, and other hydrocarbon compounds that are resistant to natural decomposition.^26,27^ These pollutants are highly toxic, tend to bioaccumulate, and can have a destructive effect on aquatic organisms and benthic ecosystems.^28,29^ In addition, hydrocarbons can quickly spread across the water surface, disrupting the oxygen balance and complicating the self-purification processes of water bodies. Their removal requires the use of materials with high sorption capacity, resistance to aggressive environments, and the possibility of subsequent recovery or disposal.

Bulk inorganic sorbents, including natural minerals such as zeolites, bentonite, sepiolite, and diatomite, are widely used to remove oil and oil products from aquatic environments due to their availability, low cost, chemical inertness, and reusability.^30,31^ Natural zeolites demonstrate pronounced efficiency in hydrocarbon retention, combining good sorption properties with a texture consisting of meso- and macropores, which promotes capillary retention of oil.^32,33^ A number of studies have shown that synthetic zeolites made from fly ash can achieve a sorption capacity of up to 1.9 g/g of diesel fuel, with sorption occurring primarily due to the physical filling of mesopores.^34,35^ In contrast, minerals such as sepiolite, montmorillonite and natural zeolite have a much lower sorption capacity (<0.2 g/g) compared to organic sorbents, such as wool fibers, which achieve sorption of up to 5.5 g/g, indicating limited penetration of oil into the pore structure of minerals.^31^ At the same time, modification of inorganic sorbents, such as organofication of clay with quaternary ammonium groups, can significantly increase hydrophobicity and resistance to degradation, which improves the structure and mechanical strength of the material while maintaining adsorption properties.^35^ Magnetic sorbents based on iron oxides, primarily Fe_3_O_4_ and γ-Fe_2_O_3_, are widely used for cleaning aqueous media from oils and petroleum products due to their magnetic separability, high regenerability, and significant sorption capacity. In contrast, α-Fe_2_O_3_-based materials, which are nonmagnetic, are often reported for comparison in terms of sorption efficiency and surface properties.^36^ A concise summary of relevant literature data, including oxide phase composition, synthesis methods, and reported oil sorption capacities, is provided in Table S1 (Supporting Information) for comparative assessment. Of particular interest are nanocomposites combining a magnetic core with porous or hydrophobic shells: thus, γ-Fe_2_O_3_/ZIF-7 demonstrates simple synthesis-modification and effective removal of crude oil with the possibility of rapid extraction with a magnet.^37^ Similarly, magnetic mixtures of Fe_3_O_4_/Bentonite, supplemented with carbon sources (rGO, MWCNTs), have a high specific surface area and a maximum sorption capacity of about 81–82 mg/g, making them well suited for cleaning water–oil emulsions.^38^ Another approach uses carbon nanocomposites: multiwalled carbon nanotubes with Fe_3_O_4_ nanoparticles (MCNTs) demonstrate a capacity of up to 6.6 g of diesel fuel per gram of sorbent and high reproducibility after regeneration.^39^ Hydrophobic Fe_2_O_3_@C nanoparticles with superoleophilicity and the possibility of multiple recovery have also been developed. Such material is quickly collected by a magnet and easily cleaned by ultrasound.^40^ Review studies emphasize that magnetic sorbents offer a unique combination of high sorption capacity, ease of magnetic separation, and resistance to repeated use, which makes them promising for emergency and routine treatment of contaminated waters.^41^ A Fe_3_O_4_/bentonite-based sorbent used in a fixed-bed column showed effective removal of oil emulsions and has potential for scalable application in wastewater treatment technologies.^42^ The rGO-Fe_2_O_3_ nanocomposite successfully removed dissolved oil from high-salt waters, where the sorption efficiency increased with increasing NaCl concentration; the adsorption capacity reached about 662 mg/g, and the process was well described by pseudo-second-order kinetics.^43^ The γ-Fe_2_O_3_/ZIF-7 composite system demonstrated high sorption capacity for oil products and hydrocarbons, with the possibility of multiple regeneration (up to 15 cycles) without significant loss of efficiency.^36^ The magnetic properties of these compounds facilitate the process of separation from purified water after treatment.^44^ One of the most accessible and technologically simple methods for obtaining such nanomaterials is solution combustion synthesis.

The choice of reducing agent and synthesis temperature plays a decisive role in determining the Fe^3+^/Fe^2+^ ratio, phase composition, and surface functionality of iron oxide materials obtained by solution combustion synthesis. Different organic fuels such as glycine, urea, citric acid, and hexamethylenetetramine provide distinct combustion environments, influencing reaction temperature, gas evolution, and reduction intensity. By systematically comparing these four agents across a range of synthesis temperatures (300–700 °C), this study aims to identify the most effective conditions for forming Fe_3_O_4_- or Fe_2_O_3_-dominated structures with optimal sorption properties. Thus, the objectives of this work were: (i) to synthesize Fe_*x*_O_*y*_-NPs samples from sediments of groundwater deironing stations using different reducing agents; (ii) to characterize the phase composition, morphology, specific surface area, wettability and zero charge point of the synthesized materials depending on the reducing agent and synthesis temperature; (iii) to determine the sorption capacity of the obtained samples for methylene blue and the oil sorption capacity (OSC) in relation to waste oil products, and to evaluate the stability of sorbents during multiple sorption-regeneration cycles; (iv) to perform statistical and correlation analysis of experimental data, to develop regression and machine learning models for predicting the oil capacity of sorbents depending on synthesis conditions.

## 2 Materials and Methods

### 2.1 Materials and Reagents

The iron source for the synthesis was derived from nitric acid leachates obtained by treating iron-rich sediments collected from iron removal facilities. The iron-containing residue had the following composition (wt %): iron—54.20, oxygen—32.82, manganese—1.05, silicon—4.46, calcium—4.07, phosphorus—2.97. The particle size of the iron-containing sediments is less than 30 μm. The leaching was done in a 20% nitric acid solution at room temperature for 60 min under mechanical stirring at 100 rpm, as detailed in ref 45. Specifics regarding the composition of the initial sediment and the leaching protocol are provided in ref 45. As fuel components for the combustion synthesis, citric acid (purity 99.5%, Sigma-Aldrich), urea (99.0%, Sigma-Aldrich), hexamethylenetetramine (HMT, 99.0%, Millipore Sigma), and glycine (99.0%, Millipore Sigma) were utilized in stoichiometric proportion to the oxidizing agent.

Spent oils from the locomotive depot were used as an adsorbate.

### 2.2 Fe_*x*_O_*y*_ NPs Synthesis

Fe_*x*_O*_y_*-based magnetic adsorption materials were synthesized via solution combustion according to the typical redox reactions between Fe­(NO_3_)_3_ (oxidizer) and organic fuels (glycine, urea, citric acid, hexamethylenetetramine). The combustion process generates both Fe_2_O_3_ and Fe_3_O_4_ phases, depending on the local reduction conditions and excess of organic fuel. Balanced reactions describing the idealized stoichiometry for partial reduction to Fe_3_O_4_ can be expressed as follows:

For Fe_2_O_3_

$$6Fe{\left({\mathrm{NO}}_{3}\right)}_{3}{+10C}_{2}H_{5}{\mathrm{NO}}_{2}{=3Fe}_{2}O_{3}{+20CO}_{2}{+14N}_{2}{+25H}_{2}O$$

$$2Fe{\left({\mathrm{NO}}_{3}\right)}_{3}{+5CH}_{4}N_{2}{O=\mathrm{Fe}}_{2}O_{3}{+5CO}_{2}{+8N}_{2}{+10H}_{2}O$$

$$6Fe{\left({\mathrm{NO}}_{3}\right)}_{3}{+5C}_{6}H_{8}O_{7}{=3\mathrm{Fe}}_{2}O_{3}{+30\mathrm{CO}}_{2}{+9N}_{2}{+20H}_{2}O$$

$$12\mathrm{Fe}{\left({\mathrm{NO}}_{3}\right)}_{3}{+5C}_{6}H_{12}N_{4}{=6Fe}_{2}O_{3}{+30\mathrm{CO}}_{2}{+28N}_{2}{+30H}_{2}O$$

For Fe_3_O_4_

$$54\mathrm{Fe}{\left({\mathrm{NO}}_{3}\right)}_{3}{+92C}_{2}H_{5}{\mathrm{NO}}_{2}{=18\mathrm{Fe}}_{3}O_{4}{+184\mathrm{CO}}_{2}{+127N}_{2}{+230H}_{2}O$$

$$18\mathrm{Fe}{\left({\mathrm{NO}}_{3}\right)}_{3}{+46\mathrm{CH}}_{4}N_{2}{O=6\mathrm{Fe}}_{3}O_{4}{+46\mathrm{CO}}_{2}{+73N}_{2}{+92H}_{2}O$$

$$54\mathrm{Fe}{\left({\mathrm{NO}}_{3}\right)}_{3}{+46C}_{6}H_{8}O_{7}{=18\mathrm{Fe}}_{3}O_{4}{+276\mathrm{CO}}_{2}{+81N}_{2}{+184H}_{2}O$$

$$54\mathrm{Fe}{\left({\mathrm{NO}}_{3}\right)}_{3}{+23C}_{6}H_{12}N_{4}{=18\mathrm{Fe}}_{3}O_{4}{+138\mathrm{CO}}_{2}{+127N}_{2}{+138H}_{2}O$$

Under strongly reducing and rapid thermal conditions, these reactions lead to a mixture dominated by Fe_3_O_4_, while under less reducing conditions or at higher temperatures, partial oxidation to Fe_2_O_3_ occurs. In this work, a 3-fold excess of organic reducing agent relative to stoichiometry was used. This approach is typical for the solution combustion method, as the use of a fuel excess relative to stoichiometric ratios increases the adiabatic combustion temperature and the rate of gas evolution (mainly CO_2_, H_2_O vapor, and N_2_), thereby enhancing the self-propagating character of the reaction and promoting pore formation in the resulting oxide structure.^46,47^ The mixture was evaporated and heated at 300, 400, 500, 600, and 700 °C for exothermic reaction initiation.

### 2.3 Fe_*x*_O_*y*_ NPs Analysis

Bruker’s D8 ADVANCE X-ray diffractometer was used for phase composition analysis of the synthesized Fe_*x*_O_*y*_-based materials. The crystalline phases identification was performed in the HighScore Plus software via Rietveld refinement.

*Determination of the specific surface* of the samples was performed by using the *BET* method with NOVA 2200 (Quantachrome, USA) using nitrogen as adsorbate.

Morphology of the synthesized Fe_*x*_O_*y*_-based magnetic adsorption materials were investigated by JSM 7600F (JEOL, Japan) field-emission scanning electron microscope (FESEM).

### 2.4 Total Static Sorption Capacity (TSSC) Test of Dissolved Organic Substances

Methylene blue, widely used in similar studies, was chosen as an organic model impurity. Methylene blue was used as a standard probe molecule to evaluate the specific surface area and the availability of active adsorption sites. It serves as a widely accepted indicator in sorbent characterization due to its cationic nature and strong interaction with oxygen-containing surface groups, rather than as a model compound for oil. This test provides a comparative measure of surface functionality among synthesized samples, whereas oil sorption capacity was determined separately (Section 2.5). Distilled water providing a concentration of 10 mg/L was used to prepare a methylene blue solution. Experiments to establish adsorption equilibrium were conducted under the following conditions: 100 mg of the sorbent sample was added to 50 mL of a methylene blue solution with an initial concentration of 10 mg/L with constant stirring. The mixtures were kept in flasks for 24 h with periodic stirring. After the adsorption process, the solid phase was separated by centrifugation. The content of methylene blue in the solutions was determined by optical density at a characteristic wavelength of 645 nm using a PV 1251C Solar spectrophotometer. Standard calibration curves were constructed based on optical absorption measurements at different dye concentrations at λ_max_ = 645 nm, after which the concentrations of the unknown solution before and after adsorption were calculated from these curves. The specific surface area *S*_sp_ (m^2^/g) was determined using the formula:

$$S_{sp}=V\times C\times N\times A_{m}/\left(m\times M\right)$$

where *V* is the volume of methylene blue solution, cm^3^; *C* is the concentration of methylene blue, g/L (mg/mL); *N* is Avogadro’s constant, *N* = 6.023 × 10^23^ mol^–1^; *A*_m_ is the area occupied by one molecule of adsorbed blue in a densely packed film on the surface of graphite, *A*_m_ = 106 × 10^–20^, m^2^; *m* is the mass of the test sample, g; *M* is the molecular mass of methylene blue, equal to 319.85 g.

### 2.5 Oil Sorption Capacity Test

Tap water was poured into a Petri dish, after which 5 mL of spent engine oil was carefully added to its surface. Then 50 mg of the studied Fe_*x*_O_*y*_ nanoparticles were applied to the oil film. A neodymium magnet was used to collect the adsorbed oil. The OSC value (in g of oil per g of nanoparticles) was calculated using the following equation:^58^

$$OSC=\left[m_{1}-\left(m_{2}+m_{3}\right)\right]/m_{3}$$

All oil sorption experiments were conducted on the water surface to simulate real conditions of oil accumulation at the air–water interface. Each test was performed in triplicate under identical conditions (50 mg of sorbent, 5 mL of spent motor oil, constant temperature, and contact time of 10 min). The reported OSC values represent the mean ± standard deviation. Consistency in sorbent distribution and oil film area was maintained to minimize the influence of particle flotation or aggregation and to ensure repeatability across different Fe_*x*_O_*y*_ compositions. The spent sorbent was regenerated by burning at 800 °C in a muffle furnace. The ash was crushed in a mortar before reusing the sorbent.

### 2.6 Point of Zero Charge

To determine pH_pzc_, a series of experiments were conducted: 50 mL of a 0.01 M NaCl solution were added to 100 mL flasks, the pH of which was preliminarily adjusted in the range of 2–10 with HCl and NaOH solutions (0.1 M). 0.1 g of a Fe_*x*_O_*y*_ sample was added to each flask, after which the suspensions were stirred for 2 h and left to infuse for 24 h. Upon completion of the experiment, the final pH value was measured using a pH meter.

### 2.7 Correlation and Statistical Analysis

Pearson correlation matrices were computed to assess relationships between input parameters and outcomes, utilizing Python-based tools. Pearson correlation coefficients were used as a basic measure of linear relationships between synthesis parameters and sorbent properties. Although this method assumes data normality, the correlations were employed here primarily for exploratory analysis given the limited data set. Data analysis was carried out using the *pandas*, *scipy*, and *statsmodels* libraries, with graphical representations generated via *seaborn*. In addition to classical statistical correlations, several machine learning algorithms (CatBoost, XGBoost, and SVR) were applied to explore nonlinear relationships between synthesis parameters and oil sorption capacity. The analysis was performed on the full experimental data set without separate training and test subsets, serving as an exploratory comparison between conventional regression and ML-based approaches rather than as a predictive model. Model performance was evaluated using 5-fold cross-validation to ensure internal consistency within the limited data set.

## 3 Results and Discussion

### 3.1 Fe_*x*_O_*y*_ NPs Characterization

The phase composition of Fe_*x*_O_*y*_-NPs obtained by solution combustion synthesis (SCS) using different reducing agents significantly depends on the nature of the fuel and the synthesis temperature. The cubic phase of magnetite (Fe_3_O_4_, space group *Fd*3̅*m*) and the hexagonal phase of hematite (α-Fe_2_O_3_, space group *R*3̅*c*) were detected in all samples, but their ratio varied widely depending on the composition (Figure 1 and Table S2). Citric acid (CA) and urea (U) showed high efficiency as reducing agents. At 300 °C, the Fe_3_O_4_ content reached 99.4% (CA) and 92.9% (U), and remained above 88% up to ∼600 °C. This is explained by the intensive formation of reducing gases (CO, NH_3_) during rapid thermolysis of these compounds, which creates strong locally reducing conditions and stabilizes the Fe^2+^-composition (magnetite).^48–50^ Glycine (G) behaves less effectively as a reducing agent. Its proportion of magnetite is low even at 400 °C (∼30%), and gradually decreases to ∼19% at 700 °C. This is due to the fact that glycine releases fewer reducing gases during combustion, which limits the reduction of Fe^3+^ to Fe^2+^, and creates a less reducing environment for magnetite formation.^51,52^ Hexamine (HMT) exhibits a temperature-dependent nature. The Fe_3_O_4_ content is ∼63% at 300–400 °C, increases sharply to ∼96.9% at 500 °C, and then decreases again at 600–700 °C. This reflects the presence of a temperature threshold at which intense decomposition of HMT sharply increases the flow of reducing gases, stabilizing Fe_3_O_4_. At higher temperatures (600–700 °C), magnetite starts to oxidize thermodynamically to α-Fe_2_O_3_, despite the presence of reducing agents.^49^ It is important to emphasize that the high proportion of Fe_3_O_4_ even at 600 °C in samples with U and CA is consistent with thermodynamic principles. Under conditions of rapid combustion and abundance of reducing gases, the magnetite phase is “fixed” since there is insufficient time for the phase transition and oxidation—this is confirmed by studies of SCS processes characterized by extreme heating rates and gas evolution.^51^ Thus, the temperature maximum of the magnetite content (400–500 °C) and the subsequent decrease in its proportion at higher temperatures are natural and are due to the balance between magnetite synthesis and the thermodynamic tendency to form hematite. Thus, when choosing CA and U as a reducing agent and the temperature regime (<500 °C), it is possible to synthesize predominantly Fe_3_O_4_ nanoparticles. If it is necessary to obtain the Fe_2_O_3_ phase, it is advisible to use less reducing or thermally stable fuels (for example, glycine), or to increase the temperature to ∼600–700 °C.

**Figure 1:**
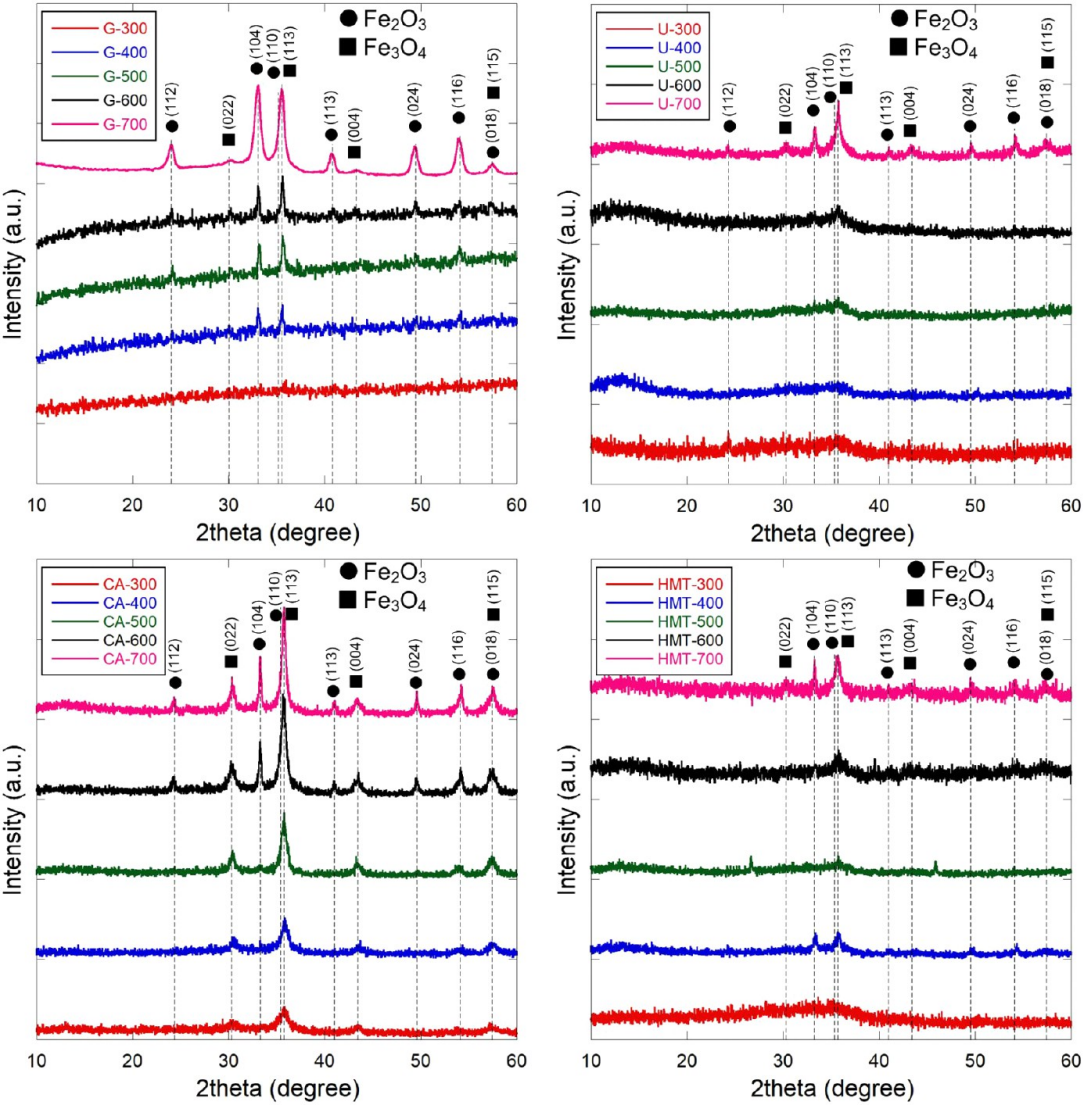
X-ray diffraction patterns of obtained Fe_*x*_O_*y*_-NPs.

FTIR spectroscopic analysis of the synthesized Fe_*x*_O_*y*_ nanoparticles revealed the presence of both characteristic residues of organic functional groups and surface compounds formed under conditions of incomplete oxidation or surface adsorption. FTIR spectra were collected for freshly synthesized samples prior to any sorption tests in order to identify surface functional groups formed during combustion. These spectra primarily reflect surface-bound organic residues resulting from incomplete oxidation of the reducing agents. Subsequent control analyses conducted after contact with water revealed no visible reduction in the absorption bands of C–H, C–O, C=O, and Fe–O. They remained unchanged. This confirms that the oxygen- and nitrogen-containing compounds identified in the fresh samples are firmly bound to the surface, rather than physically adsorbed. All spectra contain a broad band in the range of 3200–3500 cm^–1^, which is most clearly expressed in the Fe_*x*_O_*y*_-U-300 sample in the form of three overlapping maxima at ∼3250, 3350, and 3450 cm^–1^, as well as for the Fe_*x*_O_*y*_-G-300 sample. The broad absorption band at 3200–3500 cm^–1^, particularly pronounced for samples synthesized with glycine, urea, and HMT, indicates the presence of N–H stretching vibrations from surface-bound amine and amide groups (−NH_2_, – NH−). These groups likely originate from incomplete decomposition of nitrogen-containing fuels and are coordinated to Fe^3+^ sites on the particle surface. Their intensity decreases markedly above 500 °C, suggesting progressive loss of weakly bound amino fragments with increasing synthesis temperature. The presence of such bands indicates the presence of both functional residues of the reducing agent and surface-adsorbed water and a hydroxylated Fe_3_O_4_/Fe_2_O_3_ surface, which is typical for samples obtained at temperatures ≤300 °C.^53–55^ For samples obtained using citric acid, urea and urotropine, medium-intensity bands were recorded in the range of 2400–2450 cm^–1^, corresponding to asymmetric stretching vibrations of the CO_2_ molecule, or to surface carbonates (CO_3_^2–^) formed during the combustion of organic acids and nitrogen-containing reducing agents in the presence of iron oxides. These groups can form both during thermal destruction of fuel with the release of CO_2_ and as a result of subsequent carbonization of the oxide surface during cooling.^56^ The spectra of glycine, HMT and CA also contain a band at about 2850 cm^–1^, characteristic of the stretching vibrations of C–H bonds in the −CH_2_– and −CH_3_ groups, indicating residual aliphatic fragments of organic reducing agents. This confirms incomplete mineralization of the fuel under mild combustion conditions (300–400 °C) and partial organic functionalization of the iron oxide surface. The band in the 2300–2400 cm^–1^ region is due to the presence of molecular CO_2_ and/or surface carbonate groups formed during the interaction of iron oxides with atmospheric CO_2_. A characteristic feature of all samples are the bands at ∼1570 and ∼1400 cm^–1^, corresponding to asymmetric and symmetric vibrations of the carboxylate group (−COO^–^). These peaks are typical of solvated carboxylic acids or residues of decarboxylated fragments, such as citrate or iron formates. Their presence confirms partial decomposition of organic acids without complete destruction of functional groups.^57^ An additional band at 1630–1650 cm^–1^ can be attributed to the stretching vibrations of C=O (carbonyl groups) arising both from residues of aminoformyl and urea compounds and from Fe^3+^ complexes with organic ligand residues. This range can also partially overlap with vibrations of water in the bound state (H–O–H deformation modes), especially in the presence of hydrophilic surface groups. Finally, a broad band in the region of 1050–1100 cm^–1^, common to all reducing agents, indicates the presence of C–O, C–N, C–O–C and, possibly, C–OH fragments. These signals are typical for residues of carbamates, amides, products of incomplete oxidation of reducing agents, as well as surface ligand groups capable of coordinating with Fe ions. The combination of these bands indicates that as a result of exothermic synthesis at temperatures ≤500 °C, surface organic functional groups capable of influencing the hydrophilicity and adsorption properties of Fe_*x*_O_*y*_-NPs are preserved in the structure of the resulting nanoparticles. The summarized FTIR data for all synthesized samples is presented in Table S3, illustrating the dependence of key surface functional groups on the type of reducing agent and synthesis temperature Figure 2.

**Figure 2:**
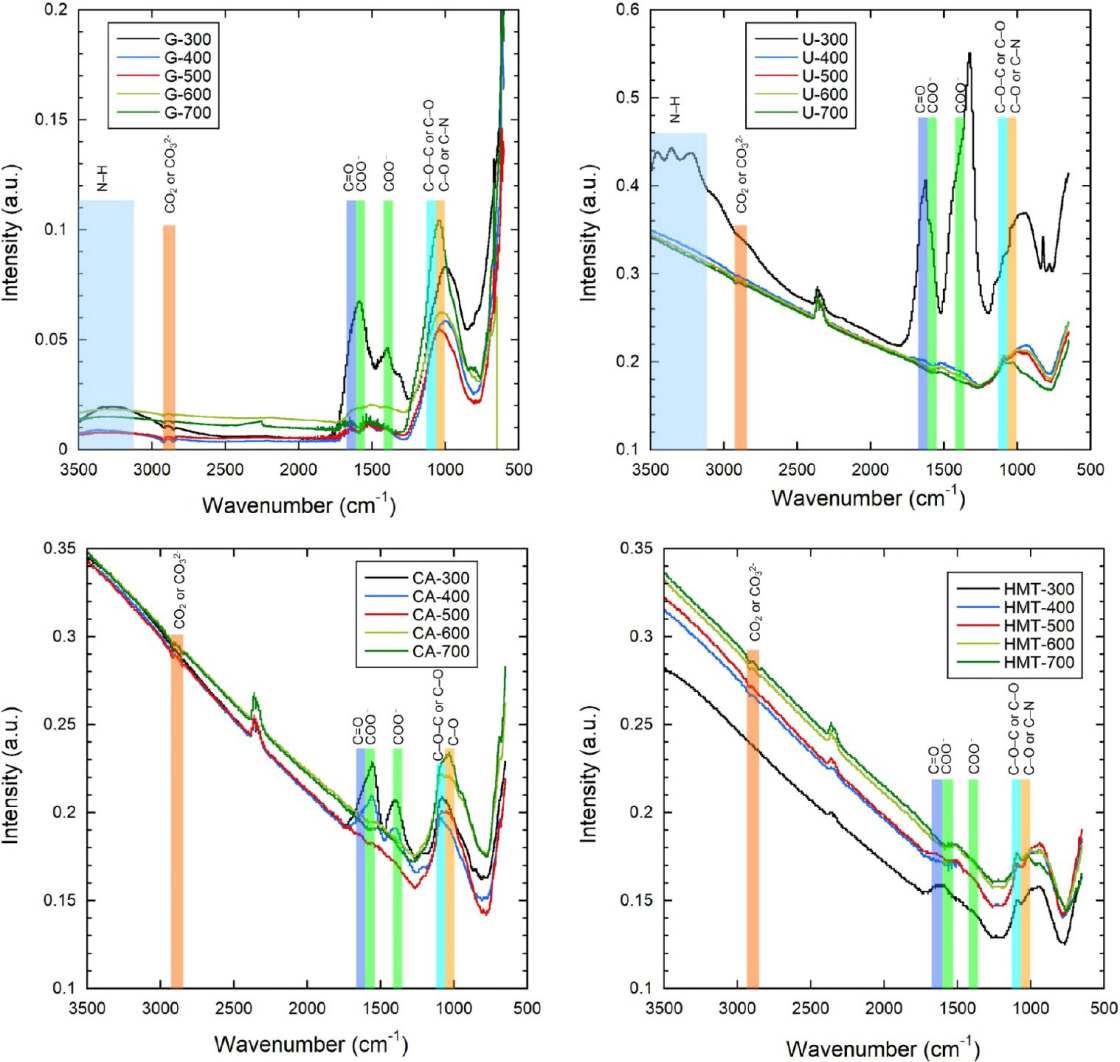
FTIR spectra of obtained Fe_*x*_O_*y*_-NPs.

Morphological analysis showed that the synthesis process mainly formed aggregates of primary nanoparticles (Figure 3 and Figure S1). When using glycine as a reducing agent, the structure acquires a well-defined porosity, characterized by a combination of micro- and nanopores, which potentially increases the availability of active centers for sorption.

**Figure 3:**
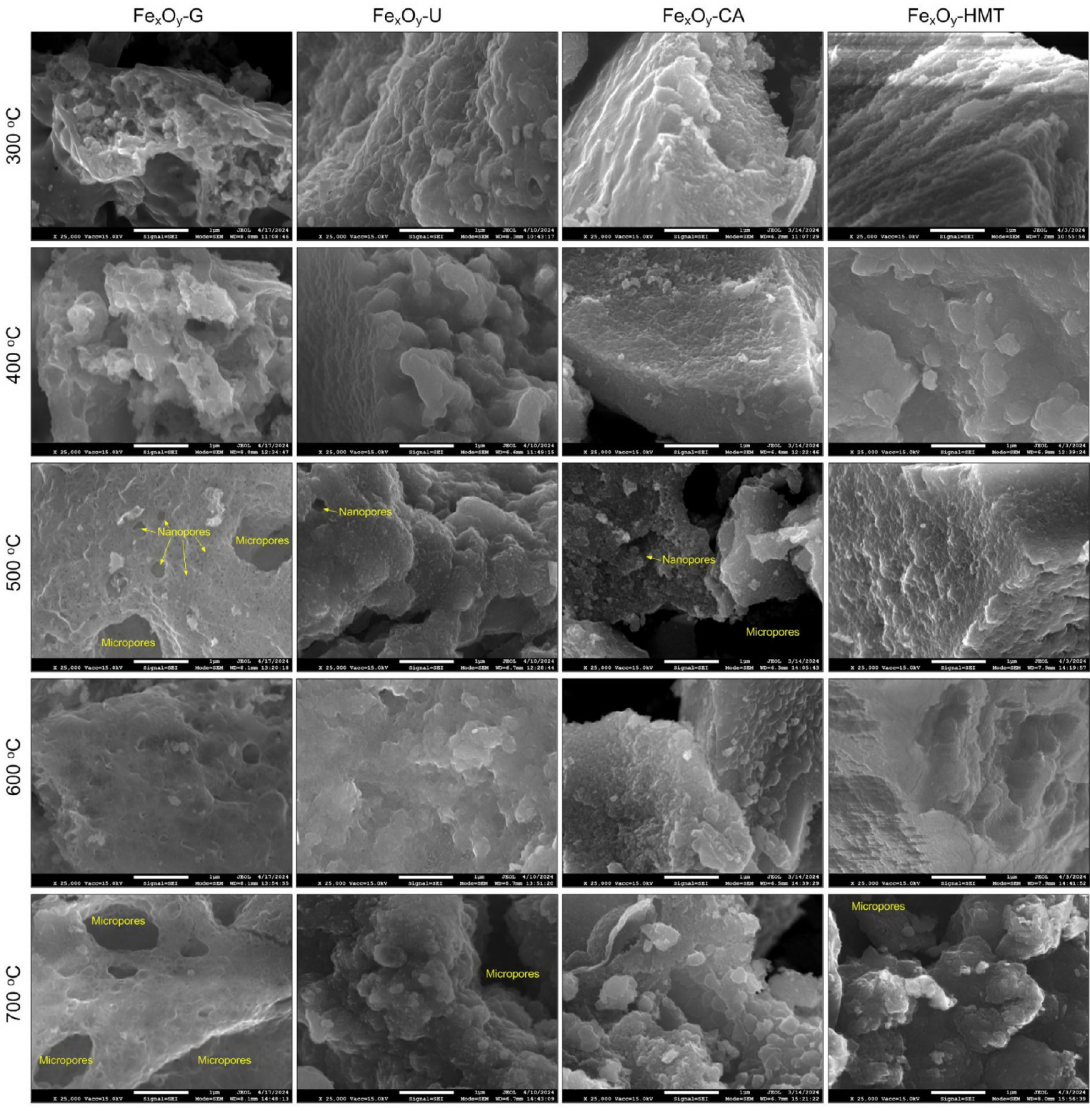
HR-SEM SED images of obtained Fe_*x*_O_*y*_-NPs (scale bar is 1 μm, magnification is ×25000).

Wetting tests showed that a drop of distilled water instantly spreads over the surface of a pressed Fe_*x*_O_*y*_ tablet, indicating its pronounced hydrophilic nature (Figure 4). This feature may be due to the presence of polar functional groups preserved on the surface as a result of using organic reducing agents, as well as to the high specific surface area and microroughness, which increase the contact area. At the same time, the samples demonstrate noticeable differences in their attitude to waste oil: depending on the reducing agent and synthesis temperature, the contact angle varied in the range from ∼60° (Fe_*x*_O_*y*_-G-500) to ∼113° (Fe_*x*_O_*y*_-HMT-700). Low angles (<70°) indicate higher lipophilicity and potentially high hydrocarbon retention capacity, while large angles (>100°) indicate the dominance of hydrophilic regions, limiting interaction with oil. Probably, such amphiphilicity is caused by a combination of a hydrophilic matrix and local lipophilic domains formed by residual carbon fragments of the reducing agent. Additionally, capillary effects in interparticle spaces and reduced surface tension of waste oil enriched with surfactant additives contribute to oil retention. Thus, the synthesized materials have double wettability—high hydrophilicity to water and the ability to adsorb hydrophobic pollutants, which is critically important for their use in wastewater treatment from petroleum products. Comparison of wettability data with oil absorption capacity (OSC, Figure 7) confirms that samples with smaller angles to oil (e.g., Fe_*x*_O_*y*_-G-500 and Fe_*x*_O_*y*_-CA-400) demonstrated maximum OSC values—up to 6.1 g/g, while materials with angles above 100° (e.g., Fe_*x*_O_*y*_-HMT-600–700) were characterized by significantly lower oil absorption capacity (<1.5 g/g). This indicates a direct dependence of the sorption capacity for oil products on the lipophilic nature of the surface, revealed in wettability experiments.

**Figure 4:**
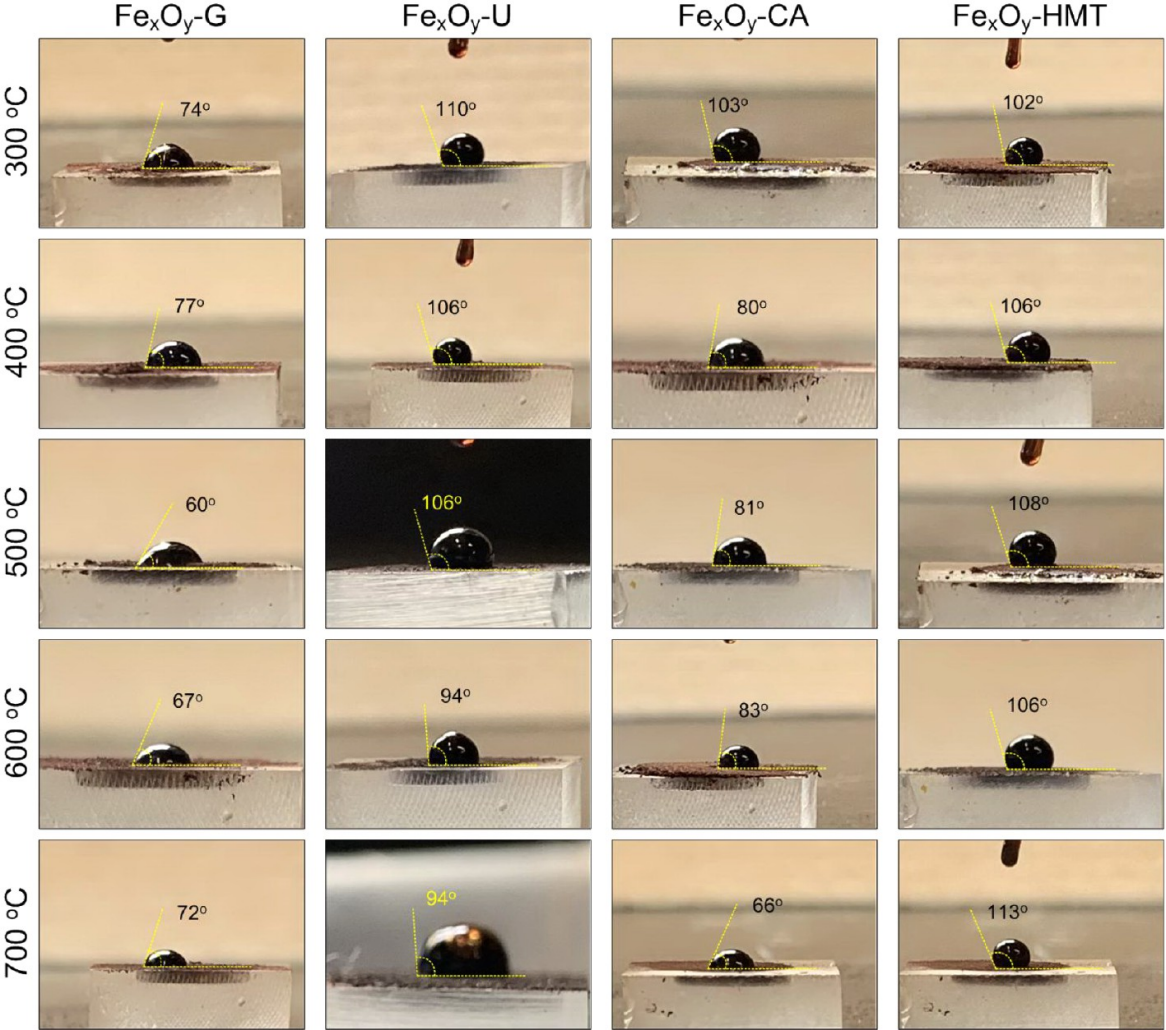
Oil wettability of obtained Fe_*x*_O_*y*_-NPs.

Measurements of the point of zero charge (pH_pzc_) showed that with increasing synthesis temperature, a gradual shift of this parameter toward the alkaline region is observed (Figure 5). For the samples obtained at 300 °C, the pH_pzc_ values were in the range of 6.8–7.9, whereas at temperatures above 600 °C they reached 8.6–8.8. A particularly noticeable increase in pH_pzc_ was recorded at 500 °C, which coincides with changes in the phase composition: according to the X-ray phase analysis data (Table 1), the proportion of magnetite (Fe_3_O_4_), which has a higher electron surface density compared to hematite (α-Fe_2_O_3_), increases in this temperature range. In addition, the FTIR results indicate a decrease in the intensity of the bands associated with carboxyl and hydroxyl groups, which indicates the burnout of organic residues and a decrease in the number of oxygen-containing acid sites. Taken together, these processes lead to a shift in pH_pzc_ toward the alkaline side due to a relative increase in the concentration of basic (Fe^2+^-containing) surface areas. Thus, the dynamics of pH_pzc_ directly reflects the balance between the Fe_3_O_4_/Fe_2_O_3_ phases and the degree of preservation of organic functional groups: at low temperatures, the surface is more oxygen-functionalized and is characterized by low pH_pzc_ values, whereas with increasing synthesis temperature, thermodynamically more stable oxides with pronounced basic reactivity are formed.

**Figure 5:**
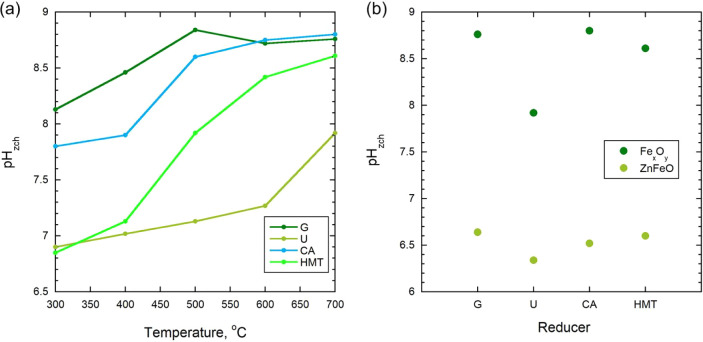
Point of zero charge (pH_pzc_) of obtained Fe_*x*_O_*y*_-NPs (a), and their comparison with ZnFeO^58^ synthesized at 700 °C (b).

**Table 1:** Composition of the Initial Fe_*x*_O_*y*_-G500 Sample, and after 1 and 5 Cycles of Regeneration

	Fe_*x*_O_*y*_-G fresh		Fe_*x*_O_*y*_-G 1 cycle		Fe_*x*_O_*y*_-G 5 cycles	
Element	at.%	wt.%	at.%	wt.%	at.%	wt.%
N	0.00	0.00	0.00	0.00	0.00	0.00
O	40.27	17.80	40.31	17.80	44.26	20.02
Si	6.31	4.90	5.16	4.00	3.66	2.90
P	3.04	2.60	3.04	2.60	2.63	2.30
S	0	0	1.24	1.10	1.77	1.60
Ca	6.95	7.70	6.78	7.50	4.77	5.41
Fe	43.42	67.00	43.47	67.00	42.92	67.77

From a practical point of view, an increase in the pH_pzc_ value means that the sorbent surface becomes less acidic and acquires a more pronounced basic character. This leads to the fact that at pH values below pH_pzc_, the surface acquires a positive charge, which increases its hydrophilicity and generally reduces the affinity for nonpolar hydrocarbons. However, in the case of spent motor oil containing oxygenated and polar components, moderate electrostatic attraction between positively charged surface sites and partially oxidized oil molecules may contribute to sorption. The highest oil sorption capacities were observed for samples with pH_pzc_ values close to the solution pH (≈6.8), where surface neutrality and enhanced hydrophobicity favor the adsorption of nonpolar oil fractions. For the sorption of organic molecules and petroleum products, this has two consequences: first, the probability of electrostatic repulsion of polar pollutants at neutral pH values decreases, and second, the proportion of hydrophobic and weakly polar areas capable of interacting with hydrocarbons increases. Thus, an increase in pH_pzc_ with an increase in the synthesis temperature indicates the formation of more stable, chemically inert and at the same time effective surfaces with respect to hydrocarbon sorption. All sorption experiments with methylene blue and oil were performed in aqueous media at natural pH of 6.8 ± 0.2, without additional adjustment.

### 3.2 Sorption Properties of Fe_*x*_O_*y*_-NPs Samples

In samples where hexamethylenetetramine was used as a reducing agent, the ion exchange capacity varied over a wider range, but decreased significantly with increasing synthesis temperature: from 4.5 mg/g at 300 °C to 2.2 mg/g at 700 °C, demonstrating a tendency for the capacity to decrease with increasing synthesis temperature. For example, the surface sorbtion capacity of a sorbent made from iron precipitate varied from 0.9 to 5.7 mg/g depending on the sample preparation method.^59^ Analysis of the total static sorption capacity (TSSC) for methylene blue and the specific surface area calculated on its basis (assuming monolayer sorption) showed significant discrepancies with the results of direct measurement of the specific surface area using the BET method (Figure 6). For most samples, the TSSC values significantly exceeded the BET indicators, especially at low synthesis temperatures. At 300 °C, the calculated data are overestimated, which can be explained by the presence of a large number of oxygen-containing functional groups on the surface (carboxyl, hydroxyl, etc.), identified by the FTIR results (Figure 2). These groups are capable of providing multilayer sorption of methylene blue, which leads to an artificial overestimation of the specific surface area in calculations from the TSSC. In the temperature range of 400–600 °C, a tendency toward a decrease in the TSSC and specific surface area is observed, which is explained by the burnout of some organic residues and partial stabilization of the oxide matrix. For samples synthesized using glycine, at elevated temperatures (400–700 °C), a better agreement is noted between the TSSC data and the results of the BET method, which is associated with a more complete removal of organic fragments and, as a result, the formation of a developed and “cleaner” surface. For materials obtained on the basis of citric acid, urea and urotropine, even at 700 °C, a significant discrepancy remains: the specific surface area according to TSSC remains 3.5–4 times higher than according to BET. This effect can be associated with the preservation of some of the surface organic groups fixed in the structure and creating additional adsorption centers, which is confirmed by the presence of carboxylate and hydroxyl bands in the FTIR spectra. Thus, when studying the sediments of chemical water purification at thermal power plants as a sorbent for oil,^60^ the specific surface varied from 23.1 to 64.7 m^2^/g depending on the sample processing temperature. For the studied soils and clays, according to the determination with methylene blue, the specific surface was in the range from 7.8 to 344.5 m^2^/g, but in most cases did not exceed 90 m^2^/g.^61,62^

**Figure 6:**
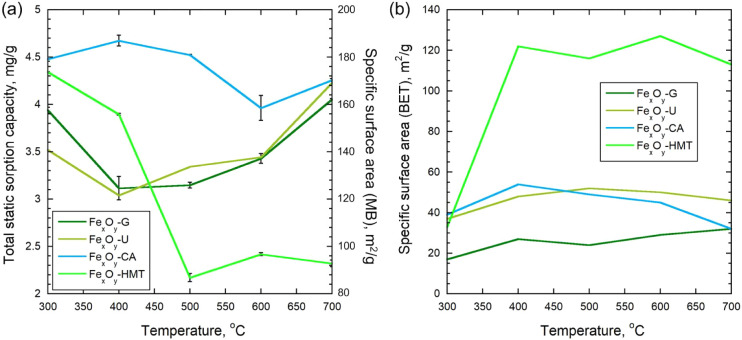
Dependence of the total static sorption capacity and the recalculated specific surface area by methylene blue sorption (a), and the specific surface area by BET—nitrogen sorption (b) vs synthesis temperature of iron-containing samples.

The results of determining the OSC showed a significant effect of both the nature of the reducing agent and the synthesis temperature (Figure 7a). The maximum OSC value reached 6.1 g/g for the samples obtained with glycine at 500 °C, which significantly exceeds the values of most other series. For the materials synthesized with citric acid, the oil capacity also remained high (up to 3.1 g/g at 700 °C). The use of hexamethylenetetramine and urea led to significantly lower OSC values (<1.6 g/g). Having carried out a comparative analysis of the OSC of industrial sorbents and the obtained Fe_*x*_O_*y*_-NPs samples, it can be concluded that the proposed sorbent (based on the iron-containing sediments of dearoning water treatment plant^63^ achieves the OSC of natural organic materials and their modifications during oil sorption. If compared with market products, the obtained values are comparable with commercial cellulose material 2.5–5 g/g,^64^ PVDF aerogel 3–7 g/g,^65^ hydrophobized hydrolytic lignins 1.6–2.5 g/g (based on fuel oil).^66^ The oil sorption process in this study is governed primarily by physical sorption mechanisms, involving both surface adsorption and capillary absorption of oil into the porous structure of the particles. Capillarity, surface roughness, and lipophilicity play dominant roles in oil uptake, while no evidence of chemical interaction between oil components and the sorbent surface was observed. The high specific surface area and interconnected pore structure facilitate the capillary retention of oil, consistent with typical behavior of oxide-based physical sorbents.

Cyclic sorption and regeneration tests showed that the OSC of the synthesized materials decreases after thermal treatment (Figure 7b). Already after the first cycle of thermal regeneration at 800 °C, the OSC decreases by 22.6% compared to the initial value. After 5 consecutive sorption-regeneration cycles, a partial restoration of the sorption capacity is observed, but it remains below the initial one by about 15.7%. According to SEM-EDS data (Figure 8 and Table 1), at each regeneration stage, a gradual increase in the sulfur content (from 0 to 1.60 wt %) is recorded, which remains in the ash from the combustion of waste oil. Oil combustion products reduce the availability of active surface centers and can partially block interparticle pores, reducing the overall oil capacity of the material while still having a sufficiently developed surface to retain oil. XRD analysis after thermal regeneration cycles (Figure 9 and Table 2) showed an increase in the proportion of hematite due to the oxidation of magnetite, which is consistent with the increase in oxygen and sulfur content revealed by EDS data (Table 1). Also, the XRD peaks shift to the right with an increase in the regeneration-sorption stages. The accumulation of sulfur-containing phases and the transition of part of Fe^2+^ to Fe^3+^ are accompanied by a decrease in the oil capacity of OSC.

**Figure 7:**
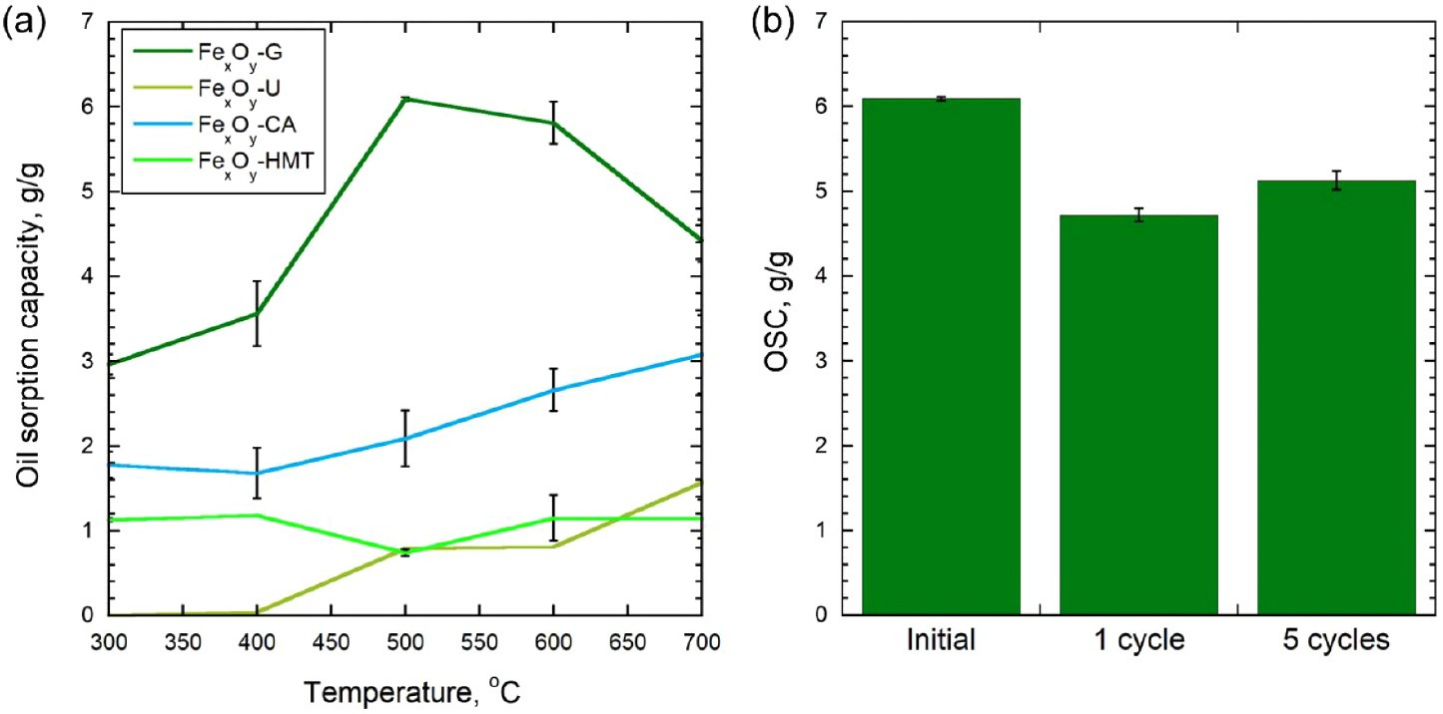
(a) OSC of obtained Fe_*x*_O_*y*_-NPs, and (b) OSC g/g of the initial Fe_*x*_O_*y*_-G-500 sample and after 1 and 5 cycles of thermal regeneration.

**Figure 8:**
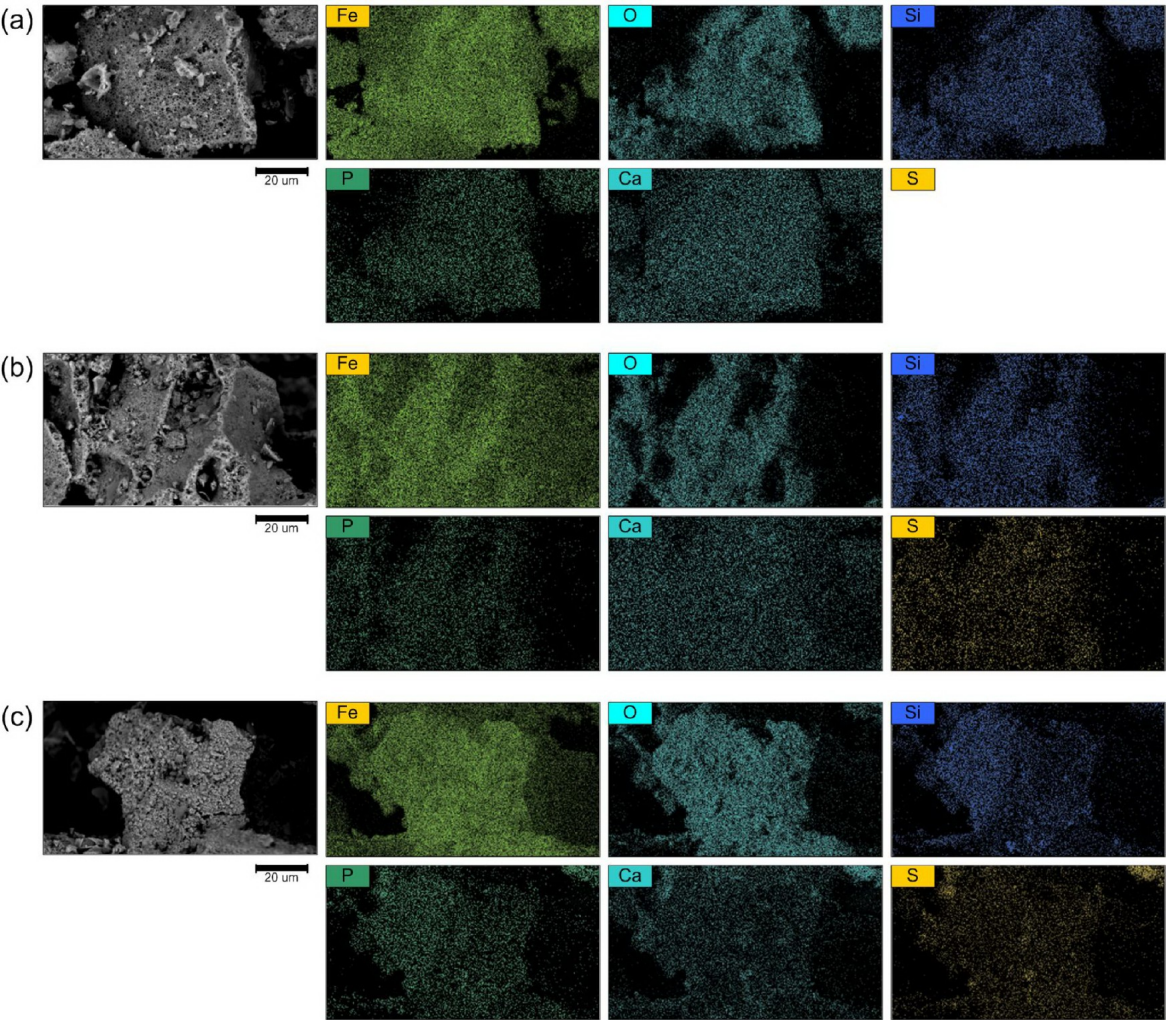
SEM-EDS images of the initial samples and after 1 and 5 cycles of thermal regeneration.

**Figure 9:**
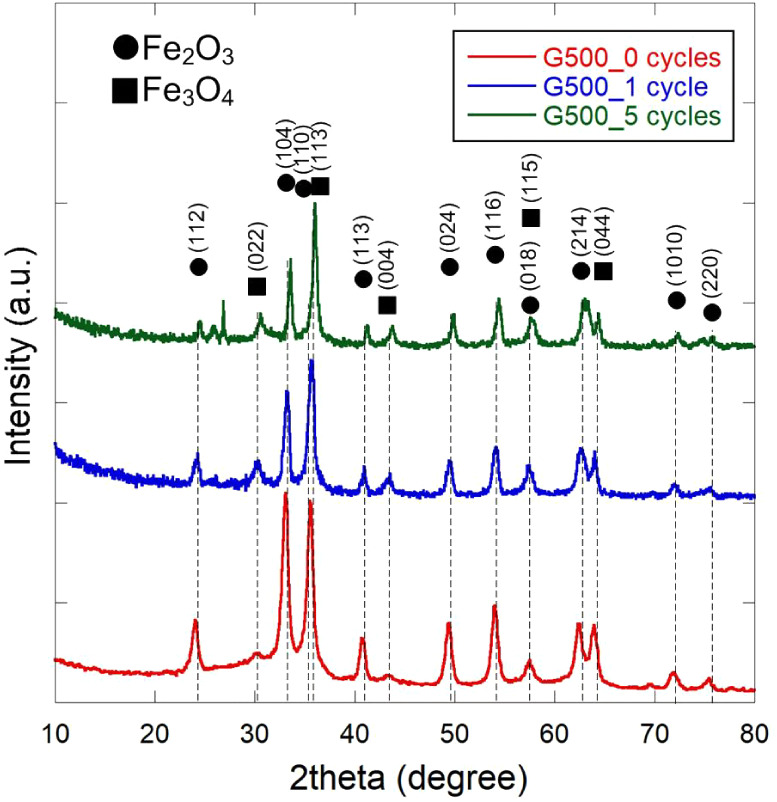
XRD diffractograms of the initial Fe_*x*_O_*y*_-G500 sample, and after 1 and 5 cycles of regeneration.

**Table 2:** Phase Composition of the Initial Fe_*x*_O_*y*_-G500 Sample, and after 1 and 5 Cycles of Regeneration

Sample	Phase	Content (%)	Crystal system	Space group
G500_0 cycles	Fe_3_O_4_	21.9	Cubic	*Fd*3̅*m*
	Fe_2_O_3_	78.1	Hexagonal	*R*3̅*c*
G500_1 cycle	Fe_3_O_4_	37.3	Cubic	*Fd*3̅*m*
	Fe_2_O_3_	62.7	Hexagonal	*R*3̅*c*
G500_5 cycles	Fe_3_O_4_	47.6	Cubic	*Fd*3̅*m*
	Fe_2_O_3_	52.4	Hexagonal	*R*3̅*c*

### 3.3 Mathematical Processing of Results

The correlation matrix reveals several strong patterns. First, along the reducing agent series G→CA→HMT→U, there is an increase in the magnetite content and a corresponding decrease in the hematite content (Table S1). With increasing synthesis temperature, the Fe_3_O_4_ content decreases, and Fe_2_O_3_ increases due to more intense thermal oxidation. Second, pH_pzc_ decreases in this reducing agent series and increases with temperature, which is consistent with the FTIR data (Figure 2). This can be attributed to the burnout of carboxyl/hydroxyl groups at 400–700 °C, which reduces the surface acidity and shifts pH_pzc_ to the alkaline region.

It is evident from Figure 10 that the oil capacity of OSC correlates positively with pH_pzc_ (PCC = 0.74) and with the content of the hematite phase (PCC = 0.82), and negatively with the proportion of the magnetite phase (PCC = −0.81). A negative correlation of OSC with the amount of the magnetite phase was also observed in the process of cyclic sorption-regeneration (Table 2). For oil wettability (W), a positive correlation is recorded with magnetite (PCC = 0.60) and negative ones with pH_pzc_ (PCC = −0.68), OSC (PCC = −0.88) and hematite (PCC = −0.60). Thus, an increase in the Fe_3_O_4_ fraction is accompanied by an increase in W (worsening of oil wettability) and a decrease in OSC, while a more “hematite” and more basic surface (higher pH_pzc_ provides a lower wettability value W and a higher oil capacity of OSC. Also, an increase in pH_pzc_ with temperature with a simultaneous increase in the hematite fraction indicates that in the range of 400–700 °C, the phase factor and surface defunctionalization act in different directions, and it is the loss of acidic functional groups (FTIR) that dominates, which is reflected in the strong OSC-pH_pzc_ relationship.

**Figure 10:**
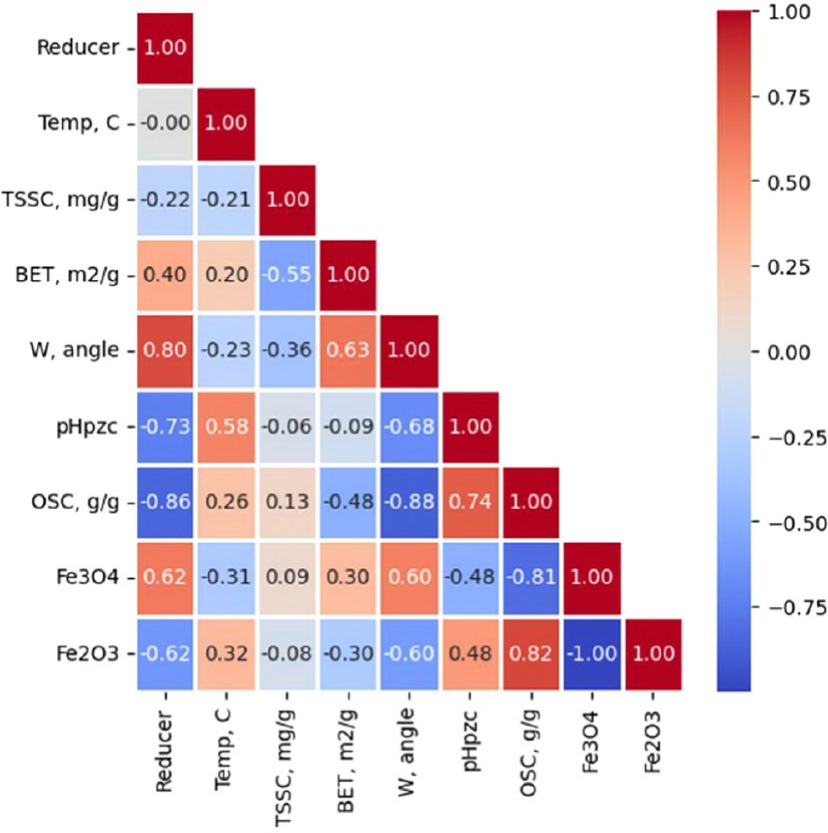
Pearson’s correlation matrix.

To describe the dependence of OSC on the type of reducing agent (Rd) and the synthesis temperature (T), both linear and nonlinear models of varying complexity were tested (Figure 11). Second-order polynomial regression made it possible to reproduce the experimental data with a fairly high accuracy of *R* ^2^ = 0.892 (Figure 11a, eq 1 and Figure 12). At the same time, the simple linear ElasticNet model gave a slightly lower level of agreement *R* ^2^ ≈ 0.80 (Figure 11e and eq 2), while its modification with polynomial features was closer to the polynomial *R* ^2^ = 0.84 (Optimal alpha: 0.18421. Optimal l1_ratio: 1.0; Figure 11d and eq 3). Similar results were shown by eLasso regression (*R* ^2^ = 0.80; Figure 11g and eq 4). Among machine learning approaches, the best results were demonstrated by the gradient boosting models CatBoost and XGBoost. The best parameters of the XGBoost model: {“colsample_bytree”: 1, “learning_rate”: 0.05, “max_depth”: 5, “n_estimators”: 500, “subsample”: 1}, which provided almost perfect agreement between the predicted and experimental values with *R* ^2^ = 1.000 (Figure 11b,f). Support Vector Machine (SVR) also showed high accuracy *R* ^2^ = 0.984, RMSE = 0.213 (Figure 11h), while Gaussian Process GPR (trained kernel GPR: 2.692 RBF (length_scale = 1.61) + WhiteKernel (noise_level = 0.194)) provided good agreement *R* ^2^ = 0.965 (Figure 11c).

1 $$OSC=3.5240-3.2833\times Rd+0.0124\times T+0.4710\times Rd^{2}-0.0007\times Rd\times T,\;R^{2}=0.8920.$$

where Rd—reducer (G—1, CA—2, HMT—3, U—4), *T*—temperature (300–700 °C)

2 OSC=2.134−1.355×Rd+0.413×T

3 $$\mathrm{OSC}=1.833-1.266\times \mathrm{Rd}+0.263\times T+0{.301\times \mathrm{Rd}}^{2}$$

4 OSC=2.13400−1.35491×Rd+0.41328×T

**Figure 11:**
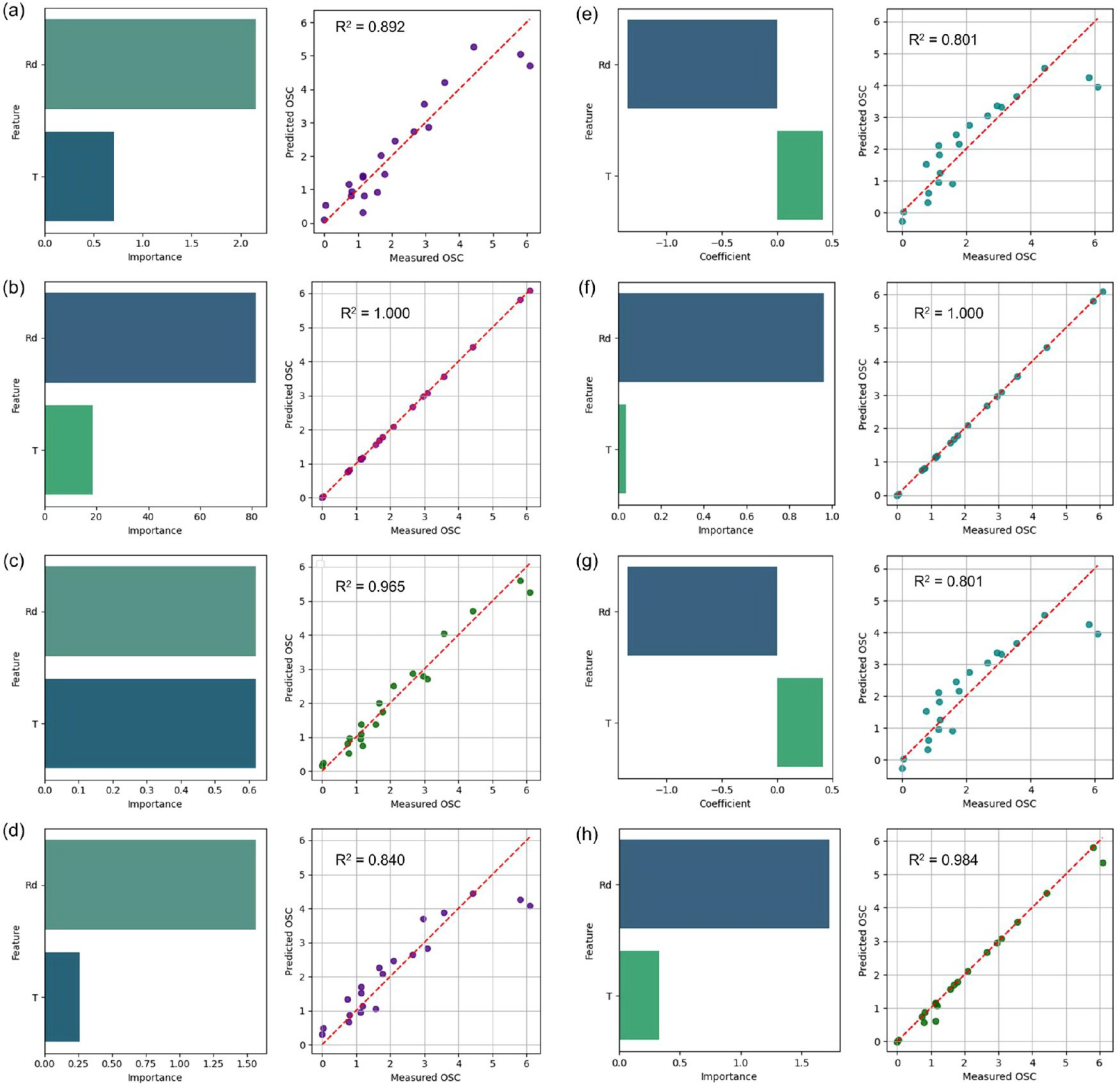
Variable importance, and plots of predicted and measured values for OSC for: polynomial equation (a), CatBoost (b), GPR (c), ElasticNet-poly (d), ElasticNet (e), XGBoost (f), eLasso (g), and SVR (h).

**Figure 12:**
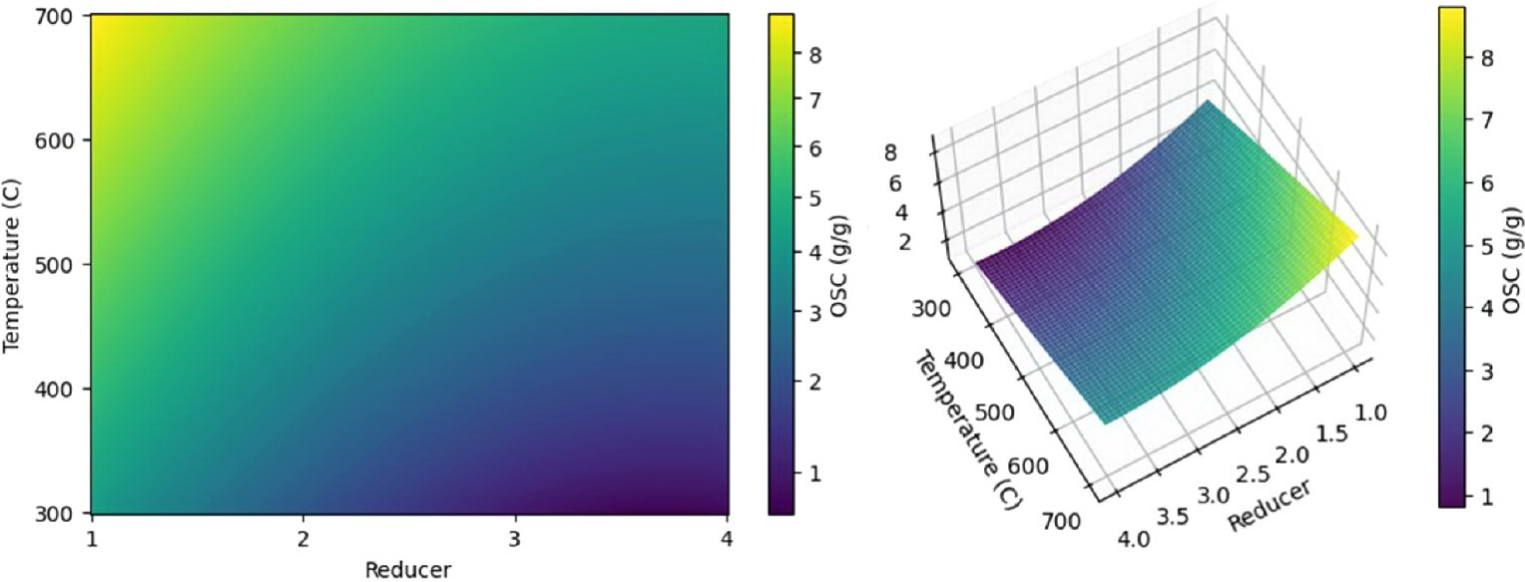
Visualization of the dependence OSC = f­(Rd; T) for a polynomial model, where for Rd: G—1, CA—2, HMT—3, U—4.

Visualization of the dependence OSC = *f* (Rd; *T*) is presented in Figure 12.

## 4 Conclusion

The results of the conducted studies of the properties of the sorbent obtained from the sediments of iron removal filter wash water purification showed the following:

- It was found that the phase composition depends on the nature of the reducing agent and the synthesis temperature. When using citric acid and urea at 300–500 °C, the proportion of magnetite (Fe_3_O_4_) reached 97–99%, while when using glycine, it did not exceed 30% and gradually decreased to ∼19% at 700 °C.
- SEM analysis revealed the aggregation of nanoparticles and the formation of sintered aggregates, while the use of glycine contributed to the formation of a more porous structure with micro- and nanopores. The pH_pzc_ values increased with increasing synthesis temperature from 6.8–7.0 (300 °C) to 8.8 (700 °C), which reflects the burnout of functional groups and an increase in the proportion of the main surface centers.
- The specific surface area calculated by methylene blue reached 186 m^2^/g, which was 3.5–4 times higher than the BET values due to multilayer sorption on oxygen-containing functional groups confirmed by FTIR. The OSC varied from <1.6 g/g (HMT, U) to a maximum of 6.1 g/g (glycine, 500 °C); for the samples with citric acid, the OSC reached 3.1 g/g at 700 °C.
- After one cycle of thermal regeneration at 800 °C, the OSC decreased by 22.6%, but after five cycles it remained only 15.7% below the initial level. SEM-EDS analysis recorded the accumulation of sulfur on the surface after each cycle, which partially explains the decrease in the sorption capacity.
- Correlation analysis showed a strong relationship between OSC and phase composition (positive with hematite and negative with magnetite), as well as with pH_pzc_. Machine learning models (CatBoost, XGBoost) provided the highest OSC prediction accuracy (*R* ^2^ = 1.0), while second-order polynomial regression reproduced experimental data with *R* ^2^ = 0.892. It should be noted that the ML analysis in this study was exploratory and not intended to produce a generalizable predictive model. The application of CatBoost, XGBoost, and SVR demonstrated their ability to capture complex dependencies even within a limited data set, highlighting the potential of ML tools for guiding the optimization of synthesis conditions in future studies.

Thus, processing of iron removal sludge into nanostructured Fe_*x*_O_*y*_ sorbents allows obtaining functional materials with high oil capacity (up to 6.1 g/g) and satisfactory stability during multiple use cycles. This confirms their potential for practical application in wastewater treatment from oil products, and also demonstrates the possibility of integrating machine learning to optimize synthesis and predict material properties.

## Supplementary Material



## Data Availability

All data used to support the outcomes of the study are included in this article.

## Notes

Table S1: literature-reported oil sorption capacities (OSC) of various iron-oxide-based sorbents; Table S2: the phase composition of synthesized Fe_*x*_O_*y*_ nanoparticles across different temperatures and reducing agents; Table S3: the FTIR spectral features and their temperature-dependent trends; Figure S1: particle size distributions for samples obtained at 500 °C (PDF)
