# Supplementary material for: Iron-Based Adsorbents Derived from Groundwater Deferrization Sludge for Spent Oil Removal from Aqueous Media
Source: ACS Environ Au. 2025 Nov 17;6(1):154–68. doi: 10.1021/acsenvironau.5c00217 (PMC12828610; doi:10.1021/acsenvironau.5c00217)
Supplement: Supplementary file 1 [file vg5c00217_si_001.pdf]

Iron-Based Adsorbents Derived from Groundwater Deferrization Sludge for Spent Oil Removal from Aqueous Media

Valentin Romanovski [1,*], Alesia Harelaya [2], Haitao Wang [3], Dmitry Moskovskikh [4]

[1] Department of Materials Science and Engineering, University of Virginia, Charlottesville, VA, 22904 USA

[2] Department of Water Supply, Chemistry and Ecology, Belarusian State University of Transport, 34 Kirova street, Gomel, 246653, Belarus

[3] College of Environmental Science and Engineering, Nankai University, Tianjin 300350, China

[4] Science and Research Centre of Functional Nano-Ceramics, National University of Science and Technology "MISIS", Moscow, 119049, Russia

* Corresponding author:

Valentin Romanovski: Department of Materials Science and Engineering, University of Virginia, Charlottesville, VA, 22904, USA; rvd9ar@virginia.edu; ORCID 0000-0003-1741-0316

Table S1
Summary of iron-oxide-based sorbents for oil removal reported in literature

Sample	Main phase	Synthesis method	OSC (g/g)	Refs
Fe_3O_4/γ-Fe_2O_3 nanoparticles	Magnetic	Co-precipitation	2.5–5.0	[36, 40, 44]
Fe_3O_4/Bentonite-rGO composite	Magnetic	Hydrothermal	0.08–0.1 g/g (oil emulsion)	[38]
γ-Fe_2O_3/ZIF-7 composite	Magnetic	Solvothermal	0.66 g/g (crude oil)	[36]
Fe_3O_4/PVDF aerogel	Magnetic	Solution casting	3–7	[64]
α-Fe_2O_3/ZnFeO nanocomposite	Non-magnetic	Combustion	0.66 g/g	[58]

Table S2
Phase composition of the obtained Fe_xO_y-NPs

Sample	Temperature, °C	Phase	Content (%)	Crystal system	Space group
Fe_xO_y-G	300	Fe_3O_4	n/a	Cubic	Fd-3m
		Fe_2O_3	n/a	Hexagonal	R-3c
	400	Fe_3O_4	30.2	Cubic	Fd-3m
		Fe_2O_3	69.8	Hexagonal	R-3c
	500	Fe_3O_4	21.9	Cubic	Fd-3m
		Fe_2O_3	78.1	Hexagonal	R-3c
	600	Fe_3O_4	19.7	Cubic	Fd-3m
		Fe_2O_3	80.3	Hexagonal	R-3c
	700	Fe_3O_4	18.9	Cubic	Fd-3m
		Fe_2O_3	81.1	Hexagonal	R-3c
Fe_xO_y-U	300	Fe_3O_4	92.9	Cubic	Fd-3m
		Fe_2O_3	7.1	Hexagonal	R-3c
	400	Fe_3O_4	91.4	Cubic	Fd-3m
		Fe_2O_3	8.6	Hexagonal	R-3c
	500	Fe_3O_4	88.4	Cubic	Fd-3m
		Fe_2O_3	11.6	Hexagonal	R-3c
	600	Fe_3O_4	90.7	Cubic	Fd-3m
		Fe_2O_3	9.3	Hexagonal	R-3c
	700	Fe_3O_4	57.9	Cubic	Fd-3m
		Fe_2O_3	42.1	Hexagonal	R-3c
Fe_xO_y-CA	300	Fe_3O_4	99.4	Cubic	Fd-3m
		Fe_2O_3	0.6	Hexagonal	R-3c
	400	Fe_3O_4	97.7	Cubic	Fd-3m
		Fe_2O_3	2.3	Hexagonal	R-3c
	500	Fe_3O_4	95.2	Cubic	Fd-3m
		Fe_2O_3	5.8	Hexagonal	R-3c
	600	Fe_3O_4	79.4	Cubic	Fd-3m
		Fe_2O_3	20.6	Hexagonal	R-3c
	700	Fe_3O_4	76.2	Cubic	Fd-3m
		Fe_2O_3	23.8	Hexagonal	R-3c
Fe_xO_y-HMT	300	Fe_3O_4	62.6	Cubic	Fd-3m
		Fe_2O_3	37.4	Hexagonal	R-3c
	400	Fe_3O_4	63.8	Cubic	Fd-3m
		Fe_2O_3	36.2	Hexagonal	R-3c

	500	Fe_3O_4	96.9	Cubic	Fd-3m
		Fe_2O_3	3.1	Hexagonal	R-3c
	600	Fe_3O_4	74.3	Cubic	Fd-3m
		Fe_2O_3	25.7	Hexagonal	R-3c
	700	Fe_3O_4	69.2	Cubic	Fd-3m
		Fe_2O_3	30.8	Hexagonal	R-3c

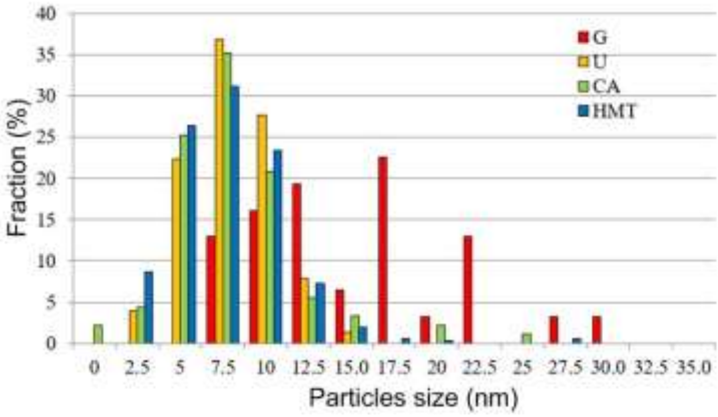

Figure S1. Particles size distribution for samples obtained at 500 C

Table S3
Main FTIR absorption bands of FexOy samples and their dependence on reducing agent and synthesis temperature

Wavenumber (cm^{-1})	Band	G	U	CA	HMT	Trend with temperature
3200–3500	–OH, N–H stretching	strong at 300–400°C	strong	medium	medium	decreases above 500°C
2850–2950	C–H (CH_2/CH_3)	medium	weak	weak	weak	disappears >500°C
1630–1650	C=O, H–O–H bending	strong	strong	medium	medium	decreases with temperature
1570–1400	–COO^- asymmetric/symmetric	strong	strong	medium	medium	decreases >400°C
1050–1100	C–O, C–N, C–O–C stretching	strong	medium	strong	medium	partially persists up to 600°C
570–580	Fe–O stretching (Fe_3O_4/ Fe_2O_3)	medium → strong	strong → very strong	strong → very strong	strong → very strong	increases with temperature
